# Crystal structure and Hirshfeld surface analysis of *N-*{[diphen­yl(vin­yl)sil­yl]meth­yl}-2-methyl­propan-2-ammonium chloride

**DOI:** 10.1107/S2056989022009112

**Published:** 2022-09-22

**Authors:** Christoph Schwab, Andreas Voss, Carsten Strohmann

**Affiliations:** a TU Dortmund University, Faculty of Chemistry and Chemical Biology, Otto-Hahn-Str. 6, 44227 Dortmund, Germany; University of Aberdeen, Scotland

**Keywords:** crystal structure, Hirshfeld surfaces, hydro­chloride, hydrogen bond, amino­methyl­silane

## Abstract

*N*-{[Diphen­yl(vin­yl)sil­yl]meth­yl}-2-methyl­propan-2-amine, C_19_H_25_NSi, is a newly synthesized secondary amino­methyl­silane that can be used, for example, to study carboli­thia­tion reactions of vinyl­silanes. Because the neutral compound did not crystallize well, the hydro­chloride salt, C_19_H_26_NSi^+^·Cl^−^, was formed, in which the two chloride ions in the asymmetric unit have crystallographic  site symmetry. An unusually long Si—C bond of 1.9117 (10) Å is observed in the cation, which may be ascribed to electronic effects due to the β N^+^ species. In the crystal, the cations and anions are linked by N—H⋯Cl hydrogen bonds to generate [001] chains. To further investigate the inter­molecular inter­actions, a Hirshfeld surface analysis was performed, which showed that H⋯H, C⋯H/H⋯C and H⋯Cl/Cl⋯H contacts contribute 70.4, 20.0 and 8.3%, respectively.

## Abstract 2

In the title hydro­chloride salt, the cation shows an unusually long Si—C bond length. In the crystal, the cations and anions are linked by N—H⋯Cl hydrogen bonds to generate [001] chains.

## 1. Chemical context

There are only a few secondary (amino­meth­yl)silanes known to date because the synthesis is not feasible due to the high energy requirement and reaction time. With the assistance of a *Finkelstein* reaction, the iodo­methyl­silane can be synthesized to enhance the reactivity and shorten the reaction time (Finkelstein *et al.*, 1910 ▸; Abele & Strohmann, 1997 ▸). However, it was possible to synthesize the (amino­meth­yl)di­phenyl­vinyl­silane **1** in an efficient way, starting from a (chloro­meth­yl)silane. Because the (amino­meth­yl)silane **1** did not crystallize well, the hydro­chloride salt **2** was formed to characterize the compound *via* X-ray diffraction. For example, the newly synthesized (amino­meth­yl)vinyl­silane **1**, C_19_H_2_5NSi, can be used for investigations of a carboli­thia­tion reaction of the silane’s vinyl group *via* lithiumalkyls. The received product can be used for the synthesis of functionalized alcohols by a *Tamao* oxidation (Tamao *et al.*, 1983 ▸)*.* The mol­ecular structure is defined by an unusually long Si—C bond, which thus favors the cleavage of this bond. Usually, the amino­methyl sidearm contains two or three nitro­gen centers and is essential for the feasibility of the reaction. It helps to break down the lithiumalkyl aggregates by forming a dative bond and also precoordinates the lithium ions, so they are in proximity to the vinyl group of the silane. Our own studies have shown that this stabilizes the transition state of the reaction, hence the activation energy of the deprotonation of the vinyl group is minimized and the reaction can be done under low temperatures and kinetic control, to prevent side reactions such as the *α*-deprotonation or polymerization (Unkelbach & Strohmann, 2009 ▸). This new (amino­meth­yl)silane **1** contains only one nitro­gen center in the sidearm and undergoes the carboli­thia­tion by a new mechanism for vinyl­silanes. This mechanism is known from stilbenes, where two lithium cations stabilize the negative charge at the anionic carbon atom. With the use of chiral ligands, the reaction can be performed under stereogenic control (Tricotet *et al.*, 2009 ▸). This opens a new field for inter­esting research in organosilicon chemistry. 
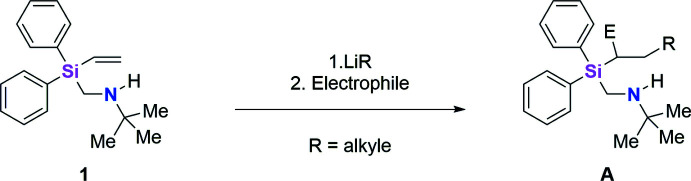
 
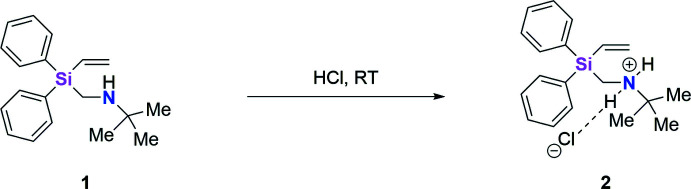


## 2. Structural commentary

**Figure 1:**
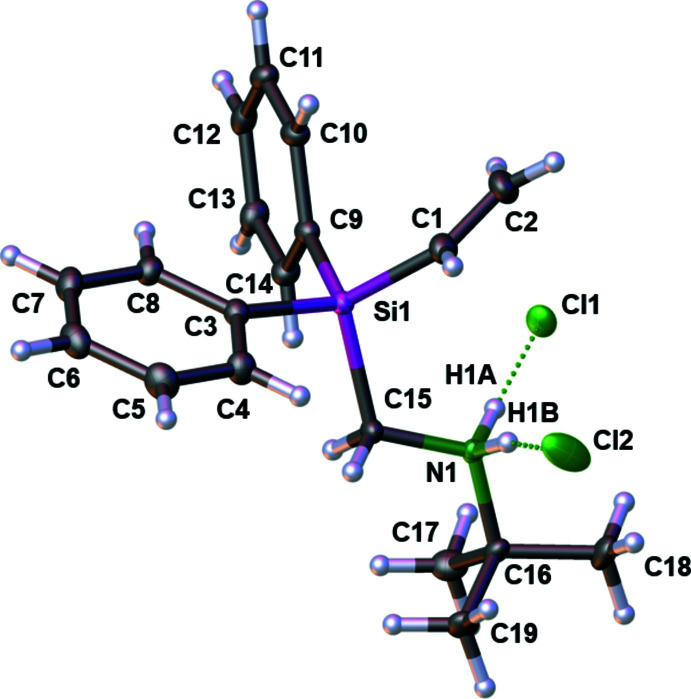
The mol­ecular structure of **2** showing 50% displacement ellipsoids. Hydrogen bonds are indicated by dotted lines.

Compound **2** crystallized in a few minutes from an aqueous 1 *M* HCl solution of **1** at room temperature as a hydro­chloride salt, C_19_H_26_NSi^+^·Cl^−^, in the form of colorless needles in the centrosymmetric space group *P*2_1_ */n*. The mol­ecular structure is illustrated in Fig. 1 ▸. Both chloride ions are located on special positions with 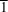 site symmetry.

The Si1—C15 bond length in the cation is 1.9117 (10) Å, which is slightly longer than the average for an Si—C bond and the Si1—C15—N1 bond angle is 116.21 (7)°. The Si—C bond lengths are normally in the range of 1.857 to 1.905 Å for C*sp^3^*—Si*X*_3_ compounds (Allen *et al.*, 1987 ▸). The extended bond length may be ascribed to the cationic nitro­gen atom in the *β*-position to the silicon atom. It increases the electronegativity, which enhances the electron-withdrawing effect of the substituted *α*-amino­functionality. This enhances the *p*-character of the Si1—C15 bond, which leads to an elongated bond (Bent, 1961 ▸). The extended Si1—C15—N1 bond angle is due to the steric demand of the *tert*-butyl group. Some further examples are given in the *Database survey* section (Kirchoff *et al.*, 2022 ▸). The angle between the C3–C8 and C9–C14 phenyl groups in **2** is 89.63 (2)°, which is caused by the steric repulsion of the aromatic hydrogen atoms. The Si1—C1 bond length is 1.8577 (11) Å and C1—C2 is 1.3293 (16) Å; the latter is positioned at the end of the default range of C*sp* ^2^—C*sp* ^2^ bonds, which lie between 1.299 and 1.328 Å.

The cationic nitro­gen center features a slightly disordered tetra­hedral geometry. The angle between the hydrogen atoms is 107.2 (13)° (H1*A*—N1—H1*B*), the angles between the C atoms and the H atoms are 107.8 (10)° (H1*B*—N1—C15) and 109.6 (9)° (H1*A*—N1—C15). Between the carbon atoms, the angle is 117.10 (7)° (C15—N1—C16). All angles vary slightly from the ideal tetra­hedron angles of 109.5°: the large C—N—C angle results from the bigger space requirement of the carbon atoms in comparison to the H atoms. The sum of angles around the nitro­gen atom is 441.7°, so the overall structure is distorted tetra­hedral. The bond length between N1 and C15 is 1.4928 (12) Å and it is 1.5330 (13) Å between N1 and C16. In the literature, C*sp^3^*—N bond lengths are in the range of 1.4816 to 1.5034 Å, so the N1—C16 bond is slightly extended.

## 3. Supra­molecular features

**Figure 2:**
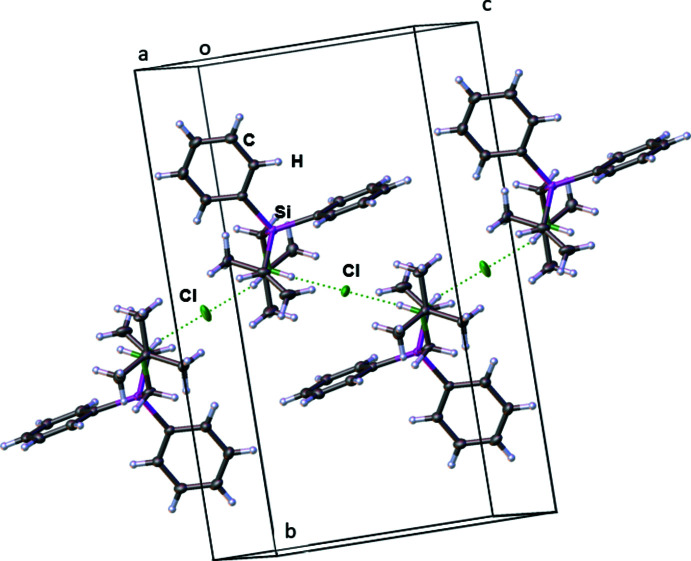
The crystal packing of compound **2** with hydrogen bonds shown as dotted lines.

**Figure 3:**
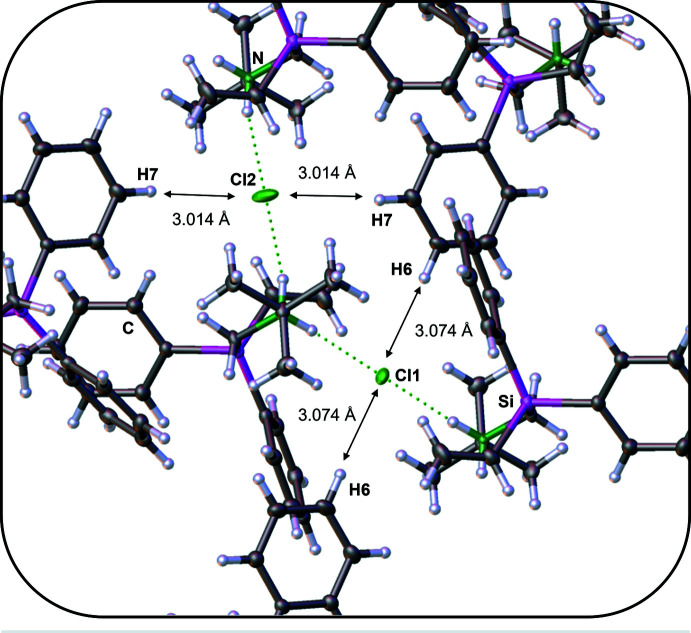
The crystal packing of compound **2** showing the C—H⋯Cl contacts.

In the extended structure of **2** (Fig. 2 ▸), the cations and anions are linked by N—H⋯Cl hydrogen bonds (Table 1 ▸) to generate chains propagating in the [001] direction. The N⋯Cl separation of 3.1184 (8) Å for the N1—H1*B*⋯Cl2 hydrogen bond is slightly longer than that for N1—H1*A*⋯Cl1 at 3.0968 (8) Å. This may be due to the different surroundings of the Cl1 and Cl2 ions in the crystal. As shown in Fig. 3 ▸, Cl1 accepts two weak, near linear hydrogen-bond contacts [C6—H6⋯Cl1: 165.82 (7)°] from the aromatic *para*-hydrogen atoms H6 with a C6⋯Cl1 distance of 4.0013 (11) Å while Cl2 accepts two weak, near linear hydrogen-bond contacts [C7—H7⋯Cl2: 165.74 (7)°] from the aromatic *meta*-hydrogen atoms H7 with a C7⋯Cl2 distance of 3.9419 (12) Å. Both contacts are formed by the same aromatic ring. The bond angle for N1—H1*B*⋯Cl2 is 164.0 (13)°, compared to 172.8 (13)° for N1—H1*A*⋯Cl1. They differ from the optimal angle of 180° because of the different surroundings in the crystal packing.

**Figure 4:**
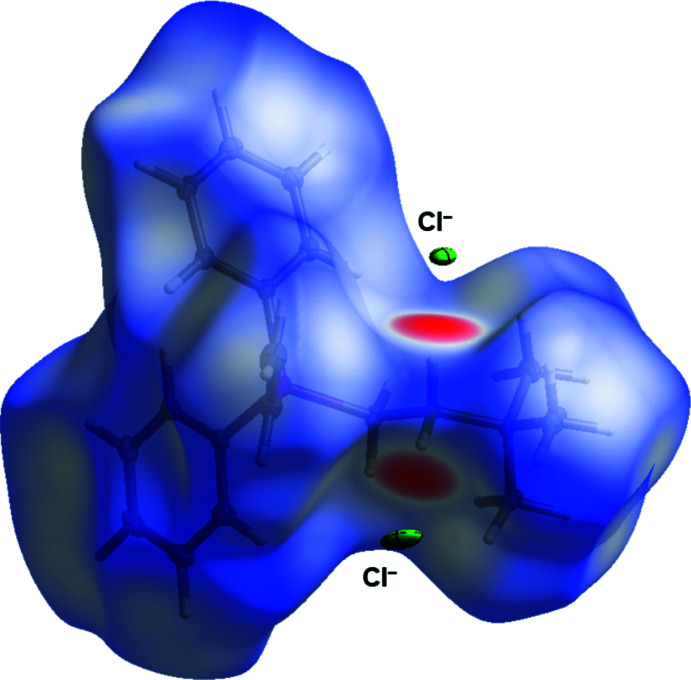
The Hirshfeld surface of compound **2** generated by *CrystalExplorer21.*

**Figure 5:**
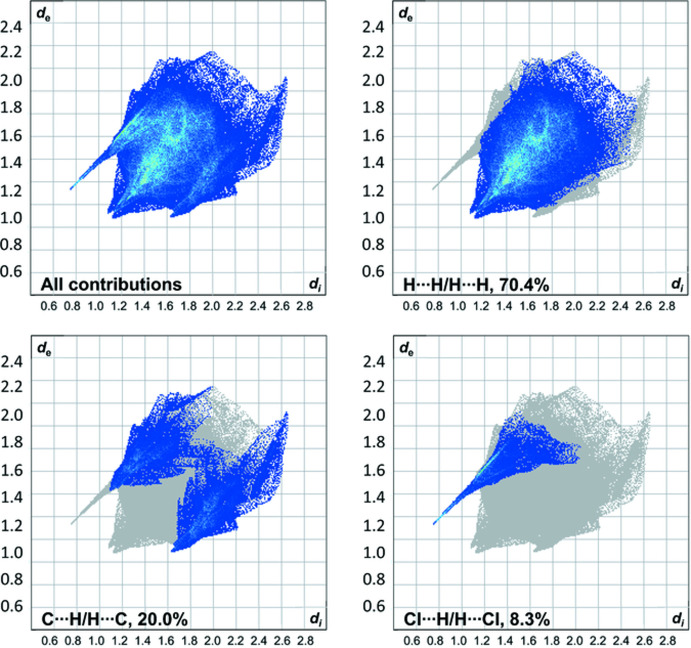
Two-dimensional fingerprint plots of compound **2** showing (*a*) all contributions in the crystal and those delineated into (*b*) H⋯H, (*c*) C⋯H/H⋯C (*d*) Cl⋯H/H⋯Cl inter­actions.

To further analyze the supra­molecular packing inter­actions, a Hirshfeld surface analysis was performed (Spackman & Jayatilaka, 2009 ▸). The Hirshfeld surface of the cation mapped over *d*_norm_ in the range from −0.54 to 1.49 arbitrary units, generated by *CrystalExplorer2021* (Spackman *et al.*, 2021 ▸; Turner *et al.*, 2017 ▸), is shown in Fig. 4 ▸. The fingerprint plots are illustrated in Fig. 5 ▸ and were also generated by *CrystalExplorer2021*. Particularly noticeable on the Hirshfeld surface are the short N—H⋯Cl contacts, which are shown in red on the potential surface, see Fig. 4 ▸. Although they represent the smallest fraction of inter­actions (8.3%), they presumably have the greatest effect on the crystal structure. The H⋯H contacts (70.4%) are the biggest fraction, but play a minor role in terms of the crystal packing. Analysis of the hydrogen-bonding network leads to the result that H1 can be assigned the graph-set symbols *D* ^1^_1_(2) and *D* ^1^_2_(3), which means that the hydrogen bond extends from N1—H1*A*⋯Cl1 to another H1*A*—N1 grouping of a neighboring mol­ecule. H2 can also be assigned *D* ^1^_1_(2) and *D* ^1^_2_(3) (Etter *et al.*, 1990 ▸). Here, the hydrogen bond extends from N1—H1*B*⋯Cl2 to another H1*B*—N1 group of a neighboring mol­ecule. These hydrogen bonds may be the reason why **2** crystallizes well compared to the neutral mol­ecule **1**.

## 4. Database survey

There are examples of crystallographically characterized structures with motifs like those in compound **2**. The following examples were found in the Cambridge Structural Database (WebCSD, May 2022; Groom *et al.*, 2016 ▸): 3,3-dimethyl-1-(4-methyl­benzene-1-sulfon­yl)-5-phenyl-1,2,3,6-tetra­hydro-1,3-aza­siline, C_19_H_23_NO_2_SSi (CSD refcode AZAFOZ; Wang *et al.* 2021 ▸), (*S*,*S*)-2-meth­oxy­methyl-1-[1-phenyl­eth­yl(dimeth­yl)sil­yl­meth­yl]pyrrolidinium iodide, C_17_H_30_NOSi^+^·I^−^ (AGILIL; Stroh­mann *et al.*, 2002 ▸), [3-(di­phenyl­phosphino)amino­(tri­phen­yl­sil­yl)methyl­idene]carbon­yl(η5-cyclo­penta­dien­yl)iron(II) tetra­fluoro­borate, C_40_H_37_FeNOPSi^+^·BF4^−^ (AMINOA; Yu *et al.*, 2010 ▸), 2-(tri­phenyl­sil­yl)pyrrolidin-1-ium chloride methanol solvate, C_22_H_24_NSi^+^·CH_4_O·Cl^−^ (LAGLUE; Bauer & Strohmann, 2017 ▸), 1-[(benzyl­dimethyl­sil­yl)meth­yl]-1-ethyl­piperidin-1-ium ethansulfonate, C_17_H_30_NSi^+^·C_2_H_5_O_4_S^−^ (WAVXAW; Kirchhoff *et al.*, 2022 ▸), 2-[ethen­yl(dimeth­yl)sil­yl]-1-[(4-nitro­phen­yl)sulfon­yl]aziridine, C_12_H_16_N_2_O_4_SSi (WOL­SEY; Astakhova *et al.*, 2019 ▸) and 1-[(benz­yl(dimeth­yl)sil­yl)meth­yl]-1-methyl­piperidin-1-ium iodide, C_16_H_28_INSi (DAFKUT; Otte *et al.* 2017 ▸).

In LAGLUE, the N—H⋯Cl hydrogen bond has a slightly longer N⋯Cl separation (3.124 Å) than compound **2**. The Si—C bond is shorter [1.905 (2) Å] and the Si—C—N bond angle is comparable [115.86 (14)°]. The lengths between the carbon atoms and the cationic nitro­gen center are similar to the corresponding bond lengths in **2** [1.498 (3) and 1.494 (3) Å].

In WOLSEY, the Si—C distance of 1.871 (4) Å is shorter than in **2** but the Si—C—N bond angle is similar [114.9 (2)°] and the C—N bond is a bit extended [1.505 (6) Å]. This could be caused by the ring strain of the aziridine ring and the electron-withdrawing effect of the (nitro­phen­yl)sulfonyl group located at the nitro­gen center. In addition, the Si—C*sp* ^2^ bond length is 1.859 (7) Å, which is only slightly longer that the value for **2**.

Finally, in AGILIL, the Si—C bond length is slightly shorter [1.907 (7) Å] and the Si—C—N bond angle is slightly extended [120.8 (4)°], which is caused by the cyclic structure of the compound. The C—N distance is equal [1.498 (8) Å] and the cyclic N—C bond lengths marginally shorter [1.509 (8) and 1.516 (8) Å], again due to the cyclic structure.

The structures of WAVXAW and DAFKUT contain a similar structure motive (Si–C–N^+^) to **2**. In WAVXAW and DAFKUT, the Si—C bond lengths are 1.9074 (11) and 1.907 (3) Å, respectively, comparable to the value in **2**. These extended bond lengths are due to the same electronic effects already described.

## 5. Synthesis and crystallization

The reaction scheme for the synthesis of compound **2** is shown in the scheme. A 1 *M* aqueous solution of HCl (0.11 mmol, 11 mL) was added to *N-*{[diphen­yl(vin­yl)sil­yl]meth­yl}-2-methyl­propan-2-amine (**1**) (0.10 mmol, 0.03 g) at room temperature. The product (**2**) was formed after five minutes as colorless needles.

## 6. Refinement

Crystal data, data collection and structure refinement details are summarized in Table 2 ▸. All hydrogen atoms except H1*A* and H1*B* were positioned geometrically (C—H = 0.95–1.00 Å) and refined using a riding model, with *U*_iso_(H) = 1.2*U*_eq_(C) for CH_2_ and CH hydrogen atoms and *U*_iso_(H) = 1.5*U*_eq_(C) for CH_3_ hydrogen atoms.

## Supplementary Material

Crystal structure: contains datablock(s) I. DOI: 10.1107/S2056989022009112/hb8033sup1.cif

Structure factors: contains datablock(s) I. DOI: 10.1107/S2056989022009112/hb8033Isup2.hkl

CCDC reference: 2207007

Additional supporting information:  crystallographic information; 3D view; checkCIF report

## Figures and Tables

**Table 1:** Hydrogen-bond geometry (Å, °)

*D*—H⋯*A*	*D*—H	H⋯*A*	*D*⋯*A*	*D*—H⋯*A*
N1—H1*A*⋯Cl1	0.923 (15)	2.179 (15)	3.0968 (8)	172.8 (13)
N1—H1*B*⋯Cl2	0.912 (15)	2.231 (15)	3.1184 (8)	164.0 (13)

**Table 2:** Experimental details

Crystal data	
Chemical formula	C_19_H_26_NSi^+^·Cl^−^
*M*_r_	331.95
Crystal system, space group	Monoclinic, *P*2_1_/*n*
Temperature (K)	100
*a*, *b*, *c* (Å)	9.7320 (11), 19.0598 (15), 10.9186 (10)
β (°)	110.526 (4)
*V* (Å^3^)	1896.7 (3)
*Z*	4
Radiation type	Mo *K*α
μ (mm^−1^)	0.26
Crystal size (mm)	0.17 × 0.10 × 0.07

Data collection	
Diffractometer	Bruker D8 Venture
Absorption correction	Multi-scan (*SADABS*; Bruker, 2016 ▸)
*T*_min_, *T*_max_	0.695, 0.746
No. of measured, independent and observed [*I* > 2σ(*I*)] reflections	58662, 7200, 5850
*R*_int_	0.045
(sin θ/λ)_max_ (Å^−1^)	0.769

Refinement	
*R*[*F* ^2^ > 2σ(*F* ^2^)], *wR*(*F* ^2^), *S*	0.037, 0.089, 1.05
No. of reflections	7200
No. of parameters	213
H-atom treatment	H atoms treated by a mixture of independent and constrained refinement
Δρ_max_, Δρ_min_ (e Å^−3^)	0.38, −0.25

Computer programs: *APEX2* and *SAINT* (Bruker, 2016 ▸), *olex2.solve* (Bourhis *et al.*, 2015 ▸), *SHELXL2014/7* (Sheldrick, 2015 ▸) and *OLEX2* (Dolomanov *et al.*, 2009 ▸).

## supplementary crystallographic information

### Crystal data

**Table:**

C_19_H_26_NSi^+^·Cl^−^	*F*(000) = 712
*M**_r_* = 331.95	*D*_x_ = 1.162 Mg m^−^^3^
Monoclinic, *P*2_1_/*n*	Mo *K*α radiation, λ = 0.71073 Å
*a* = 9.7320 (11) Å	Cell parameters from 1675 reflections
*b* = 19.0598 (15) Å	θ = 2.3–26.3°
*c* = 10.9186 (10) Å	µ = 0.26 mm^−^^1^
β = 110.526 (4)°	*T* = 100 K
*V* = 1896.7 (3) Å^3^	Block, colourless
*Z* = 4	0.17 × 0.10 × 0.07 mm

### Data collection

**Table:**

Bruker D8 Venture diffractometer	5850 reflections with *I* > 2σ(*I*)
Detector resolution: 10.4167 pixels mm^-1^	*R*_int_ = 0.045
ω and φ scans	θ_max_ = 33.1°, θ_min_ = 2.1°
Absorption correction: multi-scan (SADABS; Bruker, 2016)	*h* = −14→13
*T*_min_ = 0.695, *T*_max_ = 0.746	*k* = −29→29
58662 measured reflections	*l* = −16→16
7200 independent reflections	

### Refinement

**Table:**

Refinement on *F*^2^	Primary atom site location: iterative
Least-squares matrix: full	Hydrogen site location: mixed
*R*[*F*^2^ > 2σ(*F*^2^)] = 0.037	H atoms treated by a mixture of independent and constrained refinement
*wR*(*F*^2^) = 0.089	*w* = 1/[σ^2^(*F*_o_^2^) + (0.0302*P*)^2^ + 0.8457*P*] where *P* = (*F*_o_^2^ + 2*F*_c_^2^)/3
*S* = 1.05	(Δ/σ)_max_ = 0.001
7200 reflections	Δρ_max_ = 0.38 e Å^−^^3^
213 parameters	Δρ_min_ = −0.25 e Å^−^^3^
0 restraints	

### Special details

**Table:**

Geometry. All esds (except the esd in the dihedral angle between two l.s. planes) are estimated using the full covariance matrix. The cell esds are taken into account individually in the estimation of esds in distances, angles and torsion angles; correlations between esds in cell parameters are only used when they are defined by crystal symmetry. An approximate (isotropic) treatment of cell esds is used for estimating esds involving l.s. planes.

### Fractional atomic coordinates and isotropic or equivalent isotropic displacement parameters (Å^2^)

**Table:**

	*x*	*y*	*z*	*U*_iso_*/*U*_eq_	
Cl1	0.5000	0.5000	0.5000	0.02034 (7)	
Cl2	0.5000	0.5000	0.0000	0.03673 (12)	
Si1	0.70004 (3)	0.35895 (2)	0.32477 (3)	0.01456 (6)	
N1	0.42114 (9)	0.43180 (4)	0.22648 (7)	0.01257 (14)	
H1A	0.4520 (16)	0.4537 (8)	0.3070 (14)	0.023 (4)*	
H1B	0.4530 (17)	0.4584 (8)	0.1722 (15)	0.027 (4)*	
C1	0.78182 (12)	0.44067 (6)	0.28760 (11)	0.0215 (2)	
H1	0.7881	0.4451	0.2031	0.026*	
C2	0.83213 (14)	0.49340 (6)	0.37106 (14)	0.0298 (3)	
H2A	0.8276	0.4908	0.4564	0.036*	
H2B	0.8726	0.5337	0.3453	0.036*	
C3	0.75986 (11)	0.28120 (5)	0.25064 (10)	0.01589 (17)	
C4	0.72391 (13)	0.27683 (6)	0.11477 (11)	0.0212 (2)	
H4	0.6756	0.3151	0.0612	0.025*	
C5	0.75786 (14)	0.21724 (6)	0.05711 (11)	0.0249 (2)	
H5	0.7325	0.2152	−0.0351	0.030*	
C6	0.82860 (13)	0.16072 (6)	0.13383 (12)	0.0237 (2)	
H6	0.8505	0.1199	0.0941	0.028*	
C7	0.86709 (13)	0.16410 (6)	0.26881 (12)	0.0225 (2)	
H7	0.9162	0.1258	0.3218	0.027*	
C8	0.83342 (12)	0.22401 (5)	0.32627 (10)	0.01833 (18)	
H8	0.8609	0.2261	0.4187	0.022*	
C9	0.75476 (11)	0.34357 (5)	0.50486 (10)	0.01573 (17)	
C10	0.90359 (11)	0.34952 (5)	0.58312 (10)	0.01859 (18)	
H10	0.9723	0.3637	0.5441	0.022*	
C11	0.95274 (12)	0.33515 (6)	0.71661 (11)	0.0212 (2)	
H11	1.0538	0.3399	0.7678	0.025*	
C12	0.85334 (13)	0.31377 (6)	0.77462 (11)	0.0222 (2)	
H12	0.8863	0.3038	0.8656	0.027*	
C13	0.70528 (13)	0.30706 (6)	0.69892 (11)	0.0221 (2)	
H13	0.6372	0.2924	0.7383	0.027*	
C14	0.65699 (12)	0.32180 (5)	0.56568 (11)	0.01951 (19)	
H14	0.5558	0.3170	0.5150	0.023*	
C15	0.49188 (11)	0.36139 (5)	0.23862 (10)	0.01691 (17)	
H15A	0.4685	0.3417	0.1497	0.020*	
H15B	0.4470	0.3301	0.2866	0.020*	
C16	0.25301 (11)	0.43398 (5)	0.17306 (9)	0.01689 (17)	
C17	0.19498 (13)	0.39711 (7)	0.26910 (11)	0.0244 (2)	
H17A	0.2192	0.3471	0.2726	0.037*	
H17B	0.0882	0.4028	0.2401	0.037*	
H17C	0.2402	0.4178	0.3562	0.037*	
C18	0.21343 (15)	0.51187 (6)	0.16345 (13)	0.0292 (3)	
H18A	0.2562	0.5336	0.2499	0.044*	
H18B	0.1065	0.5171	0.1322	0.044*	
H18C	0.2522	0.5349	0.1021	0.044*	
C19	0.19597 (13)	0.39877 (6)	0.03909 (10)	0.0230 (2)	
H19A	0.2439	0.4196	−0.0176	0.035*	
H19B	0.0896	0.4056	−0.0001	0.035*	
H19C	0.2177	0.3484	0.0489	0.035*	

### Atomic displacement parameters (Å^2^)

**Table:**

	*U*^11^	*U*^22^	*U*^33^	*U*^12^	*U*^13^	*U*^23^
Cl1	0.02810 (18)	0.01878 (15)	0.01229 (13)	−0.00014 (13)	0.00477 (12)	−0.00446 (11)
Cl2	0.0490 (3)	0.0444 (3)	0.01419 (15)	−0.0234 (2)	0.00777 (16)	0.00500 (15)
Si1	0.01262 (12)	0.01221 (11)	0.01785 (12)	−0.00029 (9)	0.00410 (9)	−0.00132 (9)
N1	0.0159 (4)	0.0105 (3)	0.0101 (3)	0.0012 (3)	0.0030 (3)	0.0000 (2)
C1	0.0188 (5)	0.0170 (4)	0.0285 (5)	−0.0015 (4)	0.0080 (4)	0.0023 (4)
C2	0.0261 (6)	0.0172 (5)	0.0414 (7)	−0.0045 (4)	0.0058 (5)	−0.0013 (5)
C3	0.0149 (4)	0.0143 (4)	0.0196 (4)	−0.0002 (3)	0.0074 (3)	−0.0010 (3)
C4	0.0247 (5)	0.0187 (4)	0.0214 (5)	0.0008 (4)	0.0096 (4)	0.0005 (4)
C5	0.0305 (6)	0.0249 (5)	0.0231 (5)	−0.0014 (4)	0.0143 (5)	−0.0046 (4)
C6	0.0249 (5)	0.0189 (5)	0.0326 (6)	−0.0011 (4)	0.0168 (5)	−0.0067 (4)
C7	0.0215 (5)	0.0171 (4)	0.0312 (5)	0.0039 (4)	0.0121 (4)	0.0006 (4)
C8	0.0173 (5)	0.0171 (4)	0.0212 (4)	0.0021 (3)	0.0075 (4)	0.0002 (3)
C9	0.0136 (4)	0.0136 (4)	0.0191 (4)	0.0010 (3)	0.0046 (3)	−0.0027 (3)
C10	0.0147 (4)	0.0181 (4)	0.0218 (4)	−0.0001 (3)	0.0050 (4)	−0.0040 (3)
C11	0.0184 (5)	0.0183 (4)	0.0222 (5)	0.0030 (4)	0.0012 (4)	−0.0030 (4)
C12	0.0276 (6)	0.0163 (4)	0.0201 (5)	0.0059 (4)	0.0052 (4)	0.0005 (4)
C13	0.0233 (5)	0.0193 (5)	0.0255 (5)	0.0031 (4)	0.0107 (4)	0.0040 (4)
C14	0.0161 (5)	0.0182 (4)	0.0240 (5)	0.0010 (3)	0.0067 (4)	0.0009 (4)
C15	0.0142 (4)	0.0114 (4)	0.0221 (4)	0.0009 (3)	0.0026 (3)	−0.0024 (3)
C16	0.0148 (4)	0.0166 (4)	0.0155 (4)	0.0040 (3)	0.0006 (3)	−0.0014 (3)
C17	0.0187 (5)	0.0310 (6)	0.0255 (5)	−0.0009 (4)	0.0100 (4)	−0.0024 (4)
C18	0.0294 (6)	0.0188 (5)	0.0300 (6)	0.0112 (4)	−0.0015 (5)	−0.0022 (4)
C19	0.0223 (5)	0.0231 (5)	0.0164 (4)	0.0014 (4)	−0.0024 (4)	−0.0034 (4)

### Geometric parameters (Å, º)

**Table:**

Si1—C1	1.8577 (11)	C9—C14	1.4006 (14)
Si1—C3	1.8763 (10)	C10—H10	0.9500
Si1—C9	1.8713 (10)	C10—C11	1.3926 (15)
Si1—C15	1.9117 (10)	C11—H11	0.9500
N1—H1A	0.923 (15)	C11—C12	1.3902 (17)
N1—H1B	0.912 (15)	C12—H12	0.9500
N1—C15	1.4928 (12)	C12—C13	1.3931 (17)
N1—C16	1.5330 (13)	C13—H13	0.9500
C1—H1	0.9500	C13—C14	1.3917 (15)
C1—C2	1.3293 (16)	C14—H14	0.9500
C2—H2A	0.9500	C15—H15A	0.9900
C2—H2B	0.9500	C15—H15B	0.9900
C3—C4	1.4022 (15)	C16—C17	1.5256 (15)
C3—C8	1.4028 (14)	C16—C18	1.5281 (15)
C4—H4	0.9500	C16—C19	1.5260 (14)
C4—C5	1.3933 (15)	C17—H17A	0.9800
C5—H5	0.9500	C17—H17B	0.9800
C5—C6	1.3899 (17)	C17—H17C	0.9800
C6—H6	0.9500	C18—H18A	0.9800
C6—C7	1.3890 (17)	C18—H18B	0.9800
C7—H7	0.9500	C18—H18C	0.9800
C7—C8	1.3961 (14)	C19—H19A	0.9800
C8—H8	0.9500	C19—H19B	0.9800
C9—C10	1.4047 (14)	C19—H19C	0.9800
			
C1—Si1—C3	110.28 (5)	C10—C11—H11	120.1
C1—Si1—C9	112.03 (5)	C12—C11—C10	119.74 (10)
C1—Si1—C15	109.47 (5)	C12—C11—H11	120.1
C3—Si1—C15	104.04 (4)	C11—C12—H12	120.1
C9—Si1—C3	108.21 (4)	C11—C12—C13	119.79 (10)
C9—Si1—C15	112.51 (5)	C13—C12—H12	120.1
H1A—N1—H1B	107.2 (13)	C12—C13—H13	119.9
C15—N1—H1A	109.6 (9)	C14—C13—C12	120.10 (10)
C15—N1—H1B	107.8 (10)	C14—C13—H13	119.9
C15—N1—C16	117.10 (7)	C9—C14—H14	119.4
C16—N1—H1A	107.3 (9)	C13—C14—C9	121.26 (10)
C16—N1—H1B	107.4 (10)	C13—C14—H14	119.4
Si1—C1—H1	117.7	Si1—C15—H15A	108.2
C2—C1—Si1	124.56 (10)	Si1—C15—H15B	108.2
C2—C1—H1	117.7	N1—C15—Si1	116.21 (7)
C1—C2—H2A	120.0	N1—C15—H15A	108.2
C1—C2—H2B	120.0	N1—C15—H15B	108.2
H2A—C2—H2B	120.0	H15A—C15—H15B	107.4
C4—C3—Si1	120.21 (8)	C17—C16—N1	109.21 (8)
C4—C3—C8	117.59 (9)	C17—C16—C18	110.46 (10)
C8—C3—Si1	122.09 (8)	C17—C16—C19	111.00 (9)
C3—C4—H4	119.5	C18—C16—N1	105.20 (9)
C5—C4—C3	121.04 (10)	C19—C16—N1	109.50 (8)
C5—C4—H4	119.5	C19—C16—C18	111.30 (9)
C4—C5—H5	119.8	C16—C17—H17A	109.5
C6—C5—C4	120.35 (10)	C16—C17—H17B	109.5
C6—C5—H5	119.8	C16—C17—H17C	109.5
C5—C6—H6	120.1	H17A—C17—H17B	109.5
C7—C6—C5	119.72 (10)	H17A—C17—H17C	109.5
C7—C6—H6	120.1	H17B—C17—H17C	109.5
C6—C7—H7	120.1	C16—C18—H18A	109.5
C6—C7—C8	119.76 (10)	C16—C18—H18B	109.5
C8—C7—H7	120.1	C16—C18—H18C	109.5
C3—C8—H8	119.2	H18A—C18—H18B	109.5
C7—C8—C3	121.52 (10)	H18A—C18—H18C	109.5
C7—C8—H8	119.2	H18B—C18—H18C	109.5
C10—C9—Si1	118.64 (8)	C16—C19—H19A	109.5
C14—C9—Si1	123.67 (8)	C16—C19—H19B	109.5
C14—C9—C10	117.55 (9)	C16—C19—H19C	109.5
C9—C10—H10	119.2	H19A—C19—H19B	109.5
C11—C10—C9	121.55 (10)	H19A—C19—H19C	109.5
C11—C10—H10	119.2	H19B—C19—H19C	109.5
			
Si1—C3—C4—C5	−175.22 (9)	C9—Si1—C3—C4	175.80 (8)
Si1—C3—C8—C7	174.88 (8)	C9—Si1—C3—C8	−0.37 (10)
Si1—C9—C10—C11	−176.56 (8)	C9—C10—C11—C12	0.57 (16)
Si1—C9—C14—C13	176.07 (8)	C10—C9—C14—C13	0.42 (15)
C1—Si1—C3—C4	−61.36 (10)	C10—C11—C12—C13	−0.17 (16)
C1—Si1—C3—C8	122.47 (9)	C11—C12—C13—C14	−0.10 (16)
C1—Si1—C9—C10	−48.03 (9)	C12—C13—C14—C9	−0.04 (16)
C1—Si1—C9—C14	136.36 (9)	C14—C9—C10—C11	−0.69 (15)
C3—Si1—C1—C2	−141.05 (11)	C15—Si1—C1—C2	105.06 (11)
C3—Si1—C9—C10	73.75 (9)	C15—Si1—C3—C4	55.95 (9)
C3—Si1—C9—C14	−101.86 (9)	C15—Si1—C3—C8	−120.22 (9)
C3—C4—C5—C6	−0.07 (18)	C15—Si1—C9—C10	−171.88 (7)
C4—C3—C8—C7	−1.38 (16)	C15—Si1—C9—C14	12.52 (10)
C4—C5—C6—C7	−0.76 (18)	C15—N1—C16—C17	65.05 (11)
C5—C6—C7—C8	0.50 (17)	C15—N1—C16—C18	−176.39 (9)
C6—C7—C8—C3	0.59 (17)	C15—N1—C16—C19	−56.69 (11)
C8—C3—C4—C5	1.12 (16)	C16—N1—C15—Si1	−172.72 (6)
C9—Si1—C1—C2	−20.47 (12)		

### Hydrogen-bond geometry (Å, º)

**Table:**

*D*—H···*A*	*D*—H	H···*A*	*D*···*A*	*D*—H···*A*
N1—H1*A*···Cl1	0.923 (15)	2.179 (15)	3.0968 (8)	172.8 (13)
N1—H1*B*···Cl2	0.912 (15)	2.231 (15)	3.1184 (8)	164.0 (13)
